# My Creed

**Published:** 1892-08

**Authors:** Hudson Tuttle


### MY CREED.

To pass along the way of common life,
With patience doing what I find to do,
Avoiding hatred and the breath of strife,
To right and justice striving to be true.

To bear the burdens that are mine to bear,
Accept the duties of my lot and place,
The world against the right to calmly dare,
An error from my path strive to efface.

I have a creed, then this is creed of mine,
For I regard it as of smallest worth.
Though I receive and every creed combine,
If doing, in my soul receives not birth.

My brothers may believe or not believe ;
May bow at Christian or at Moslem shrine,
I will not at their adoration grieve,
Nor ask that their belief be like to mine.

Above the tempest of conflicting creeds,
A Pharos light burns through the cloudy nights,
A noble purpose answers all our needs,
And surely guides us to the heavenly heights.

—HUDSON TUTTLE.

